# Enhanced Thermal Conductivity of Free-Standing Double-Walled Carbon Nanotube Networks

**DOI:** 10.1021/acsami.3c09210

**Published:** 2023-10-27

**Authors:** Jake Dudley Mehew, Marina Y. Timmermans, David Saleta Reig, Stefanie Sergeant, Marianna Sledzinska, Emigdio Chávez-Ángel, Emily Gallagher, Clivia M. Sotomayor Torres, Cedric Huyghebaert, Klaas-Jan Tielrooij

**Affiliations:** † Catalan Institute of Nanoscience and Nanotechnology (ICN2), BIST and CSIC, Campus UAB Bellaterra, Barcelona 08193, Spain; ‡ imec vzw, Kapeldreef 75, Leuven 3001, Belgium; § ICREA, Passeig Lluís Companys 23, Barcelona 08010, Spain; ∥ Department of Applied Physics, TU Eindhoven, Den Dolech 2, Eindhoven 5612 AZ, The Netherlands

**Keywords:** carbon nanotubes, thermal conductivity, Raman thermometry, lithography, pellicle, extreme ultraviolet

## Abstract

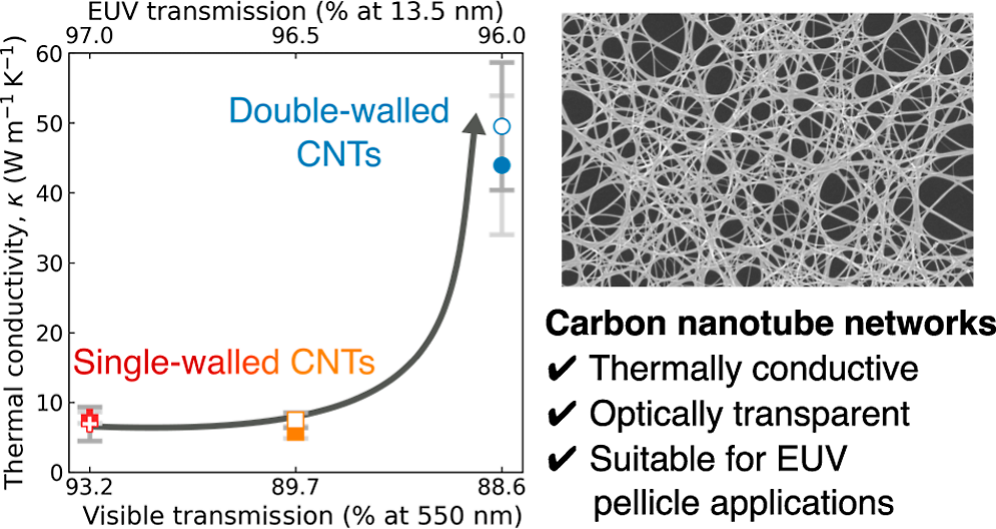

Nanomaterials are driving advances in technology due to their oftentimes superior properties over bulk materials. In particular, their thermal properties become increasingly important as efficient heat dissipation is required to realize high-performance electronic devices, reduce energy consumption, and prevent thermal damage. One application where nanomaterials can play a crucial role is extreme ultraviolet (EUV) lithography, where pellicles that protect the photomask from particle contamination have to be transparent to EUV light, mechanically strong, and thermally conductive in order to withstand the heat associated with high-power EUV radiation. Free-standing carbon nanotube (CNT) films have emerged as candidates due to their high EUV transparency and ability to withstand heat. However, the thermal transport properties of these films are not well understood beyond bulk emissivity measurements. Here, we measure the thermal conductivity of free-standing CNT films using all-optical Raman thermometry at temperatures between 300 and 700 K. We find thermal conductivities up to 50 W m^–1^ K^–1^ for films composed of double-walled CNTs, which rises to 257 W m^–1^ K^–1^ when considering the CNT network alone. These values are remarkably high for randomly oriented CNT networks, roughly seven times that of single-walled CNT films. The enhanced thermal conduction is due to the additional wall, which likely gives rise to additional heat-carrying phonon modes and provides a certain resilience to defects. Our results demonstrate that free-standing double-walled CNT films efficiently dissipate heat, enhancing our understanding of these promising films and how they are suited to applications in EUV lithography.

## Introduction

The integration of information and communication technology into society is growing exponentially with sectors, including smart devices, wearables, transportation, and more. An integral part of this trend is the miniaturization of components as well as the incorporation of new functionalities such as mechanical flexibility. In this regard, nanomaterials offer distinct advantages over traditional materials, such as bulk silicon, due to their reduced dimensionality. One type of nanomaterial that has received significant interest is networks of one-dimensional materials, such as nanowires or nanotubes made from materials such as silicon, SiC, and bismuth.^1−4^ Several works suggest exploiting the relatively low thermal conductivity of these nanomaterials toward thermoelectric applications.

Carbon nanotubes (CNTs) are a nanomaterial with excellent electrical and mechanical properties that make them a promising material for flexible electronics, with device applications in chemical and biological sensing, transparent electrodes, and displays, some of which are already available in the market.^5^ Heat dissipation is a critical issue for both flexible and nonflexible electronics. In the former, typical polymer substrates such as poly(ethylene terephthalate), PET, have comparatively low thermal conductivities <0.2 W m^–1^ K^–1^.^6^ For applications such as interconnects in microelectronics, CNT films are already important due to their favorable thermal properties.^7^ For another application, namely, EUV pellicles, the CNT thermal properties and ability to withstand the heat associated with EUV exposures become vital, next to high EUV transparency and mechanical strength of the CNT membrane.^8^

Advances in EUV lithography are driving the miniaturization of integrated circuits to the 5 nm process level and beyond.^9,10^ Crucial to this advancement is the development of pellicles that protect the photomasks from damage during lithography. EUV pellicles were initially fabricated with polysilicon (pSi), but their low EUV transmission (83%) motivated the search for other material systems.^11^ In addition, the thermal conductivity (κ) of pSi films is rather low, with κ = 14 W m^–1^ K^–1^ for a 1 μm thick film.^12^ For thinner films, the increased boundary scattering further reduces this value. This is true for both pSi and single-crystal silicon, where the thermal conductivity drops 15-fold as the thickness decreases from 1 μm to 9 nm.^13^ Other materials include thin graphite or silicon nitride films. Thin graphite films grown by chemical vapor deposition have a higher thermal conductivity (κ ≈ 700 W m^–1^ K^–1^)^14^ but a smaller EUV transmission (70%),^15^ which limits their applicability for EUV lithography. Amorphous silicon nitride has a lower thermal conductivity (2.7 W m^–1^ K^–1^)^16^ and a relatively high EUV absorption (14% for a 16 nm membrane), which leads to overheating and failure of these films under EUV powers >80 W.^17^ To be thermally stable, a pellicle should have a high EUV transmission and a high thermal conductivity as this will lead to the smallest temperature increase due to EUV absorption. Different materials can handle different heat loads, but as a starting point, the EUV transmission should be >90%^18^ and the thermal conductivity should ideally be higher than that of current materials that are considered for EUV pellicles, which means above 10 W m^−1^ K^−1^. None of the above candidates satisfy these requirements. However, several novel pellicle materials have been proposed to support future EUV scanners with higher source powers and associated technology nodes, in particular, metal silicide composite material and CNT-based pellicles.^19^

Free-standing CNT films consisting of a randomly oriented CNT network have been found to be a promising pellicle material candidate for high EUV source powers, exhibiting high EUV transmission and thermal and mechanical stability as well as offering a range of additional advantageous material properties.^19,20^ Recent demonstrations have shown that CNT pellicles have minimal impact on wafer imaging during exposure in an EUV scanner.^21^ The stability of free-standing CNT films under high EUV powers motivates the study of their thermal properties. Individual CNTs are among the best conductors of heat with room-temperature thermal conductivities greater than 2000 W m^–1^ K^–1^.^22,23^ This is reduced by one or more orders of magnitude in films and sheets made from CNTs as the tubes form a percolation network (κ = 0.5–200 W m^–1^ K^–1^).^24−30^ Notably, there is an absence of experimental studies on the thermal properties of double-walled CNT networks.^31^ We also note that in most studies, the CNT films are supported on a substrate. Disentangling the intrinsic and extrinsic factors contributing to thermal transport is not trivial. Therefore, determining the intrinsic thermal conductivity of free-standing CNT films, in particular, double-walled networks, is both technologically relevant and fundamentally interesting.

In this paper, we investigate the thermal conductivities of free-standing CNT films using Raman thermometry.^32^ Using the temperature-dependent frequency of the G^+^ Raman mode, we determine the thermal conductivity at temperatures between 300 and 700 K of single-walled and double-walled CNT films (SWCNT and DWCNT, respectively). The reported values are smaller than those for individual nanotubes, highlighting the role played by intertube junctions in thermal transport of CNT networks.^33−39^ Interestingly, DWCNT films are roughly seven times as thermally conductive as SWCNT films with comparable EUV transmission. This suggests that the second wall prevents additional phonon-defect scattering, which would otherwise impede thermal transport, and provides a parallel channel for heat conduction. Importantly, these films efficiently dissipate heat, making them suitable for applications such as EUV pellicles, interconnects in integrated circuits, and components in flexible electronics.

## Experimental Section

**Figure 1:**
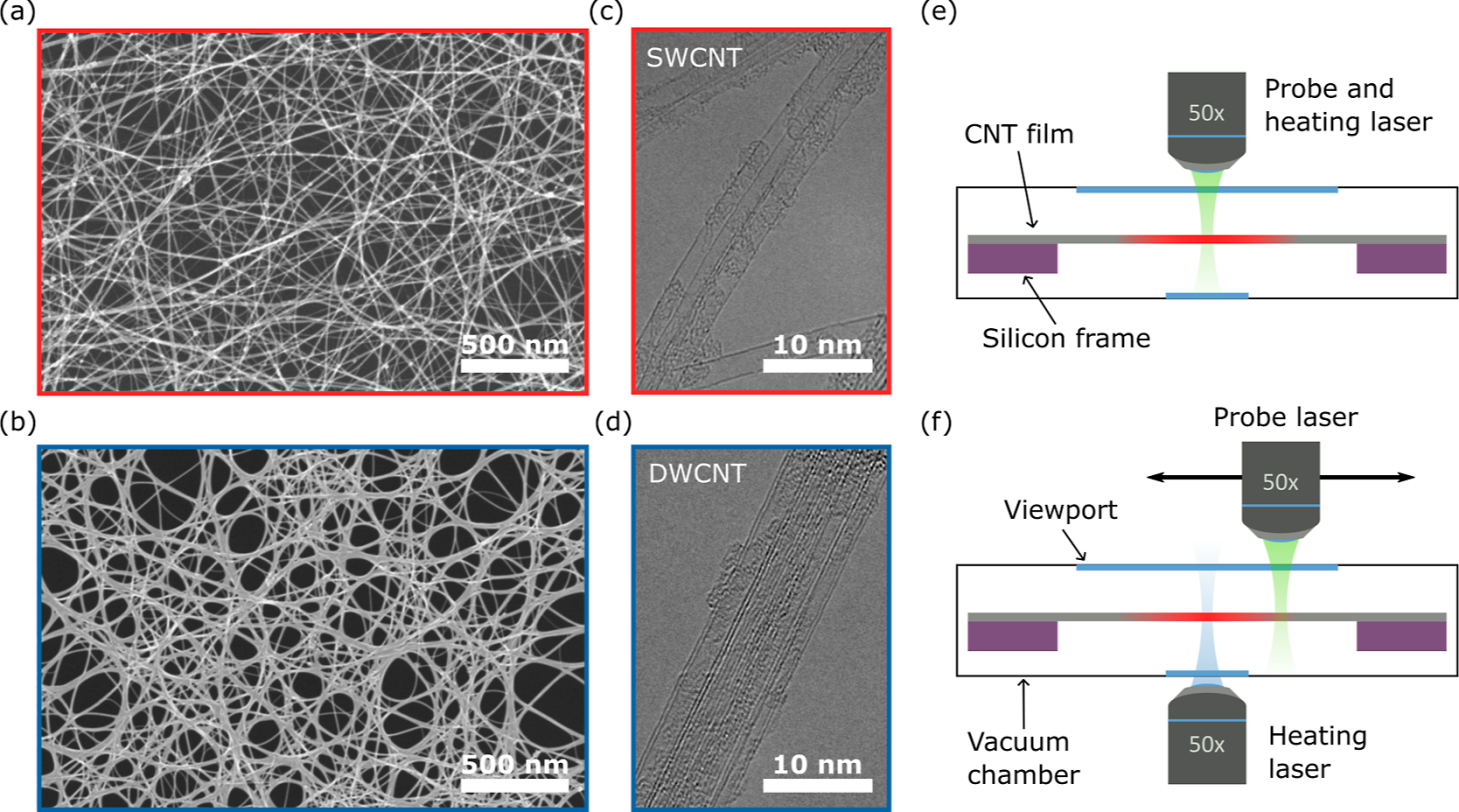
Samples and measurement configurations. (a,b) SEM images of (a) single- and (b) double-walled carbon nanotube (SWCNT and DWCNT) networks. They are composed of randomly oriented CNT bundles with numerous intertube junctions. (c,d) TEM images of (c) SWCNT and (d) DWCNT bundles. (e,f) Schematics of the (e) one- and (f) two-laser Raman thermometry techniques (1LRT and 2LRT). The absorbed laser power locally heats the pellicle (red hot spot), softening the phonon modes. This results in a redshift of their Raman frequency, which the probe laser probes using Raman scattering spectroscopy. In 1LRT (e), a single laser serves as both the heater and thermometer, while 2LRT (f) employs a separate heating laser. In 2LRT, the scanning probe laser maps the temperature profile.

We investigated two types of free-standing CNT films containing either SWCNTs or DWCNTs, which were fabricated as follows.^20,40^ SWCNTs were collected directly after their synthesis in a floating catalyst chemical vapor deposition reactor onto a microporous filter. Random CNT films were further transferred to a support frame and densified with a solvent.^66^ DWCNT films were first assembled onto a filter from a CNT dispersion by means of vacuum filtration. After filter removal, the DWCNT film floating in solution was transferred onto a support frame to form the pellicle membrane.^40^Figure 1 shows the SEM images of (a) SWCNT and (b) DWCNT networks. Many individual CNTs form these networks and randomly connect at multiple junctions. Catalyst particles from growth are present in both types of CNT films, which increase their EUV absorption. High-resolution TEM images of individual CNTs and bundles are shown in Figure 1c,d.

We study three samples: one DWCNT film (D1) and two SWCNT films (S1 and S2). Table S1 summarizes the thickness and optical properties of these films. We used atomic force microscopy to determine the film thickness, see Note S3. The two SWCNT films have thicknesses of 10.9 nm (S1) and 26.6 nm (S2), controlled by varying the CNT collection time. For the thicker film, the visible transmission at 550 nm is lower (89.7% compared with 93.2%). Notably, although the DWCNT film is the thinnest (8.6 nm), it has the lowest transmission (88.6%). This is due to the additional wall in the nanotube structure, which increases absorption.

To study the thermal properties of free-standing CNT films, we use two variations of Raman thermometry. This noninvasive, all-optical technique, also referred to as optothermal Raman spectroscopy, is widely used to measure the thermal conductivity of nanomaterials.^32,41−43^ The first variation of the technique, one laser Raman thermometry (1LRT), uses a continuous-wave laser beam both as a thermometer and heat source, see Figure 1e. The calibration of the thermometer is conducted by leveraging the temperature dependence of a Raman mode using a low-power laser to avoid self-heating effects. Once calibrated, power-dependence measurements are performed by maintaining a constant environmental temperature. In order to be as accurate as possible, we measure the incident power exactly in the sample plane using calibrated power meters that are mounted directly in the Raman thermometry setup. The local temperature is indirectly monitored via the laser-induced frequency shift using the thermometer calibration. The absorbed laser light generates an excited electronic population that relaxes by transferring energy to the lattice in the form of phonon heat.

The noninvasive aspect of the Raman thermometry technique is particularly advantageous to study free-standing thin films, as no additional fabrication steps are necessary, thereby allowing a direct and reliable determination of the intrinsic thermal properties of materials such as CNT pellicles.

To estimate the thermal conductivity, we consider that the heat flux is directed radially outward from the laser spot. In other words, the heat carried by phonons diffuses from this hot spot. This is valid assuming that the material has uniform absorption in the out-of-plane direction and isotropic in-plane thermal conductivity. We neglect radiative cooling based on calculations in ref (44) for similar measurements on a material system with similar in-plane conductivity and temperatures. We obtain the thermal conductivity (κ) by solving the 2D heat equation for a free-standing membrane^41,42^
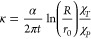
1where *t* is the film thickness, *r*_0_ is the laser spot size, and α is a geometric term (≈1). χ_T_ and χ_P_ are the changes in Raman mode frequency as a function of temperature (χ_T_) and absorbed laser power (χ_P_), respectively. *R* is the thermal decay length, which is the length over which the system returns to ambient temperature. Details on how we obtained the absorbed laser power are given in Note S4.

In the second variant—two-laser Raman thermometry (2LRT)—the addition of a second focused laser allows for the decoupling of the heat source and temperature probe, Figure 1f.^32^ In this configuration, the probe laser maps the temperature profile by obtaining spatially dependent Raman spectra with respect to a fixed pump laser that locally heats the sample. In this way, this technique directly maps the thermal field distribution. We obtain the thermal conductivity using the following expression, which is valid in a regime where the thermal field decays linearly in ln *r*(32)
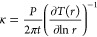
2where *P* is the absorbed laser power and *t* is the sample thickness. κ is obtained from the slope of the temperature (*T*) against ln (*r*). In contrast to 1LRT (eq 1), no prior knowledge about the geometry of the experimental setup, such as the spot size or thermal decay length, is required for 2LRT (eq 2).

In order to use Raman thermometry techniques, we first identify a temperature-dependent Raman mode in the samples, which we use for temperature calibration. The Raman spectra were collected using a Horiba T64000 Raman spectrometer in a single-grating configuration with a 2400 g/L grating. All Raman measurements were carried out using a diode laser (λ_0_ = 532 nm, Cobolt) focused via a high NA objective to a 1/e spot size of *r*_1/e_ = 0.88 μm. In 1LRT, the Raman laser spot acts both as a heater and as a local temperature probe; see Figure 1e. For 2LRT, the heat source was a 405 nm laser placed below the sample (*r*_1/e_ = 0.79 μm), see Figure 1f. A variable-temperature cryostat (Linkam) evacuated to a pressure *P* < 5 × 10^–3^ mbar housed the samples. The vacuum conditions both protected the samples from atmospheric contamination and eliminated unwanted heat dissipation processes due to convection and thermal conduction to the air. We used vacuum conditions for both types of Raman thermometry measurements.

**Figure 2:**
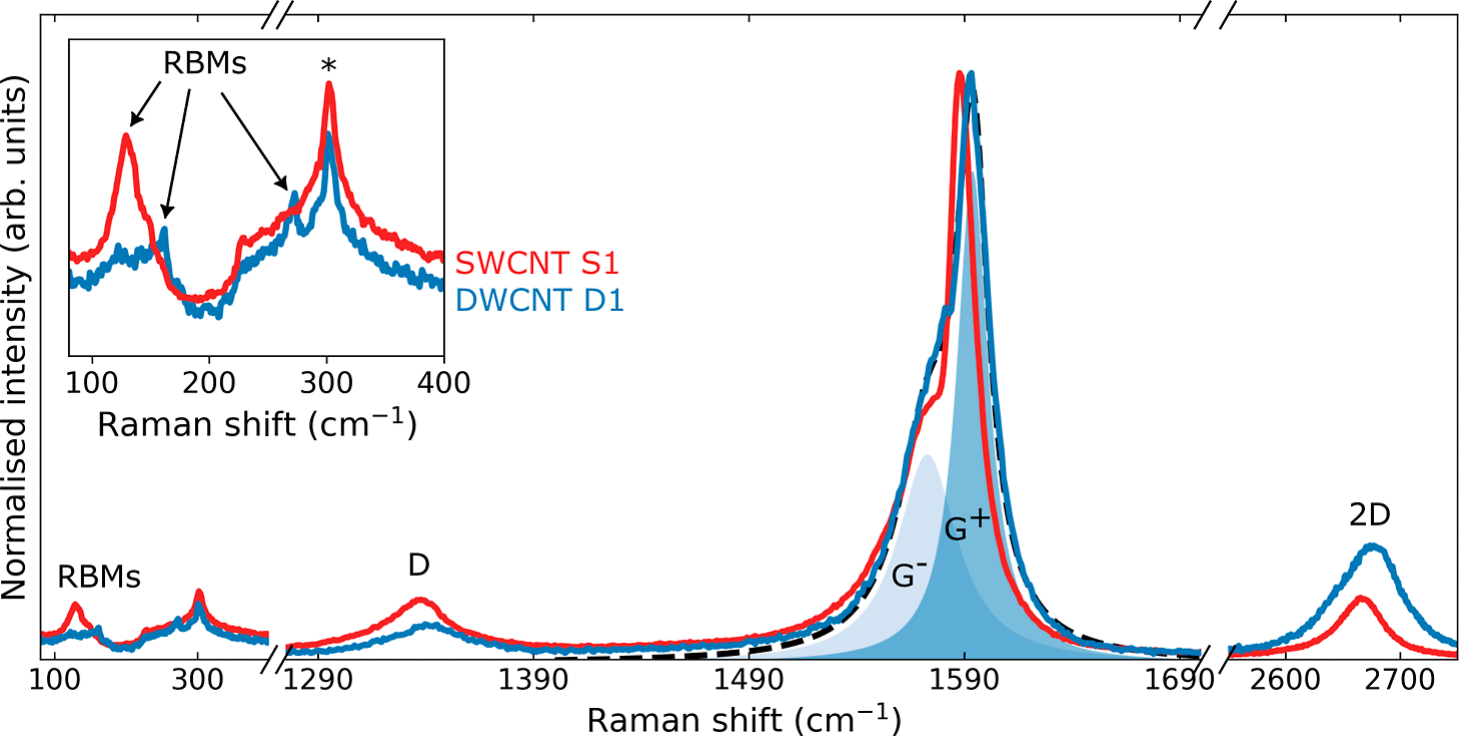
Raman spectra of CNTs. Spectra of SWCNT S1 (red) and DWCNT D1 (blue) networks normalized to the intensity of the peak around 1590 cm^–1^. Multiple peaks arise from the D, G, 2D, and RBM phonon modes, with the latter shown in the inset. In CNTs, the G peak splits into two components (G^–^, G^+^), each described by a Lorentzian profile (shaded blue), see the main text, allowing us to accurately determine the peak position. The black dashed line is the sum of these profiles. DWCNT D1 has a smaller D peak and greater 2D peak intensity than those of SWCNT S1. This suggests that DWCNT D1 has fewer defects and a higher electronic quality. To avoid laser-induced heating, we obtain the spectra on the part of the CNT films supported by the silicon substrate. The asterisk (*) indicates a peak arising from silicon.

Figure 2 shows the Raman spectra of the DWCNT films D1 and SWCNT S1 films. The spectra consist of multiple peaks in the spectral range from 100 to 3000 cm^–1^ consistent with the observed data.^45−49^ We normalize each spectrum to the intensity of the most prominent peak around 1590 cm^–1^, which comes from G band phonons, common to all graphitic compounds. This peak contains two components, one located near 1590 cm^–1^ (G^+^) and the other around 1570 cm^–1^ (G^–^), that arise from the longitudinal (G^+^) and circumferential (G^–^) motions of the carbon atoms in the nanotube, respectively. The G^+^ mode frequency is particularly sensitive to doping,^50^ strain,^51,52^ and temperature^53,54^ while being independent of tube diameter (*d*_t_). On the other hand, the G^–^ frequency is dependent on *d*_t_.^47^ Hence, the G^+^ mode is the most suitable for Raman thermometry measurements. In the results that follow, we will use the frequency of the G^+^ Raman mode as a local temperature probe. The high-resolution spectrometer used in this work and the high accuracy of the peak fitting algorithms result in a spectral resolution approaching 0.05 cm^–1^. This would allow for the detection of small changes in temperature (∼2 K). Ultimately, we are limited by the spatial inhomogeneity of the G^+^ mode (∼0.8 cm^–1^, see Note S1), which corresponds to a temperature uncertainty of Δ*T* ∼ 30 K.

Having identified a suitable temperature-dependent Raman mode, we briefly turned our attention to the other features in the Raman spectra. The peaks around 1340 and 2680 cm^–1^ arise from the D and 2D phonon modes (see Figure 2), which are associated with defects and electronic quality, respectively.^55^ The low intensity of the D peak, relative to the G, highlights the low defect density of these films.^56,57^ For the DWCNT film, the higher (lower) intensity of the 2D (D) mode compared to that of the SWCNT film suggests that the DWCNT sample has fewer defects and better electronic quality.^58^ The low-frequency radial breathing modes (RBMs) found between 100 and 500 cm^–1^ arise from the coherent, radial, out-of-plane motion of carbon atoms and are unique to CNTs.^49^ In the inset of Figure 2, we identify a single peak in the case of SWCNT films and two for DWCNT films. The presence of these RBMs confirms the single- and double-walled nature of the CNT films, in agreement with the TEM images (Figure 1c,d).

## Results

**Figure 3:**
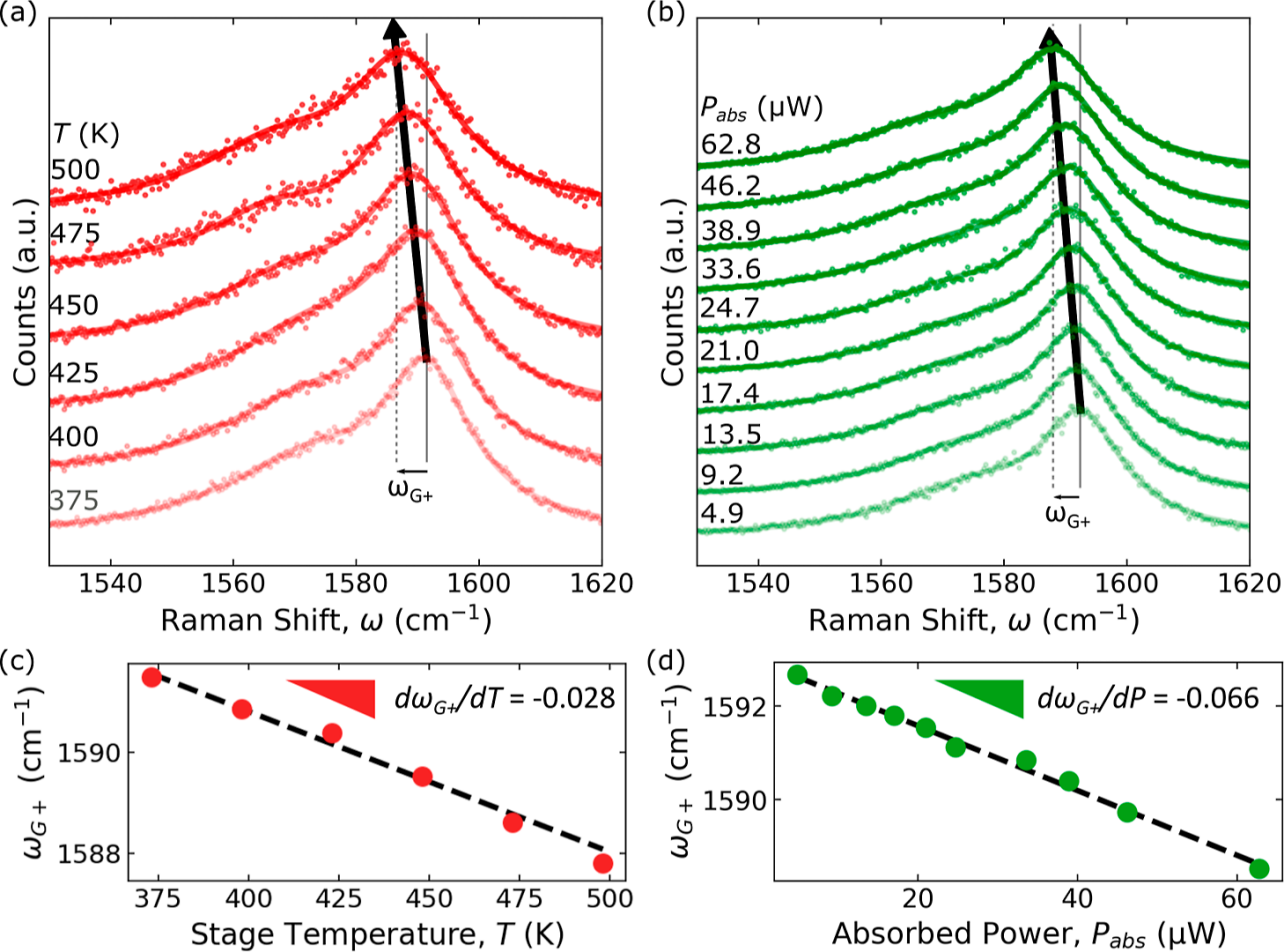
Temperature and laser power dependence of DWCNT D1 Raman modes. (a,b) Raman spectra of the free-standing DWCNT film (D1) obtained as a function of the stage temperature (a) and the laser power (b), which heat the sample globally and locally, respectively. The solid and dashed lines highlight the G^+^ frequency at ambient and elevated temperatures, while the black arrow highlights the shift in peak frequency. The higher the temperature, the greater the shift. (c,d) Shift in G^+^ mode frequency as a function of temperature (c) and absorbed laser power (d). Here, the fitting error is smaller than the symbol size. The gradient of a linear fit (black dashed line) provides the calibration for temperature and laser-induced heating, which are shown in (c,d).

Using the temperature-dependent G^+^ Raman mode, we now calibrated the frequency shift while heating the sample. Figure 3 shows the stage-temperature and laser-power dependence of the Raman spectra of the free-standing DWCNT films, which correspond to global and local heating of the CNT film, respectively. As the stage temperature increases, the G modes shift to lower frequencies, as shown in Figure 3a.

Increasing the temperature by 100 K red-shifts the G modes by approximately 3 cm^–1^. This is due to the softening of the G phonon modes.^53,54^ By tracking the G^+^ peak frequency (ω_G_^+^) as a function of stage temperature, we obtain the temperature coefficient χ_T_ = dω_G^+^_/d*T* = −0.028 cm^–1^ K^–1^, Figure 3c. This agrees with previous reports of DWCNT agglomerates suspended in methanol^59^ and SWCNT films supported on SiO_2_.^60^ Here, the low-power laser acts as a nanoscale temperature probe.

Having verified that the G^+^ peak frequency represents the lattice temperature of the free-standing CNT network, we now study the dependence on absorbed laser power; see Figure 3b. Increasing the laser power results in a redshift of the G^+^ mode frequency because the absorbed optical energy heats the pellicle, leading to the softening of the phonon modes. Figure 3d shows the dependence of ω_G^+^_ on the absorbed laser power. The power coefficient is χ_P_ = dω_G^+^_/d*P* = −0.066 cm^–1^ μW^–1^. This value is greater than that in previous reports of SWCNT films supported on SiO_2_ (χ_P_ = −0.01 cm^–1^ μW^–1^).^30^ The reason that we observe a larger shift, and thus a larger temperature increase, for the same power is that in their case, heat sinking into the SiO_2_/Si substrate was present, meaning that they required a larger power to reach the same temperature increase, which corresponds to a smaller χ_P_. For free-standing CNT films, the local temperature depends exclusively on the ability of the CNT network to dissipate heat. We obtain the thermal conductivity using the temperature and power coefficients in eq 1. Taking the thermal decay length (*R*) to be the distance over which the temperature decays from 80 to 20%, we calculate κ = 49.5 ± 9.1 W m^–1^ K^–1^ for the DWCNT sample using *R* ≈ 100 μm. We note that if we take the decay length to correspond to the 90 to 10% decay length, we would find *R* ≈ 130 μm. This would result in a thermal conductivity of 52 W m^−1^ K^−1^, which is within the reported uncertainty.

**Figure 4:**
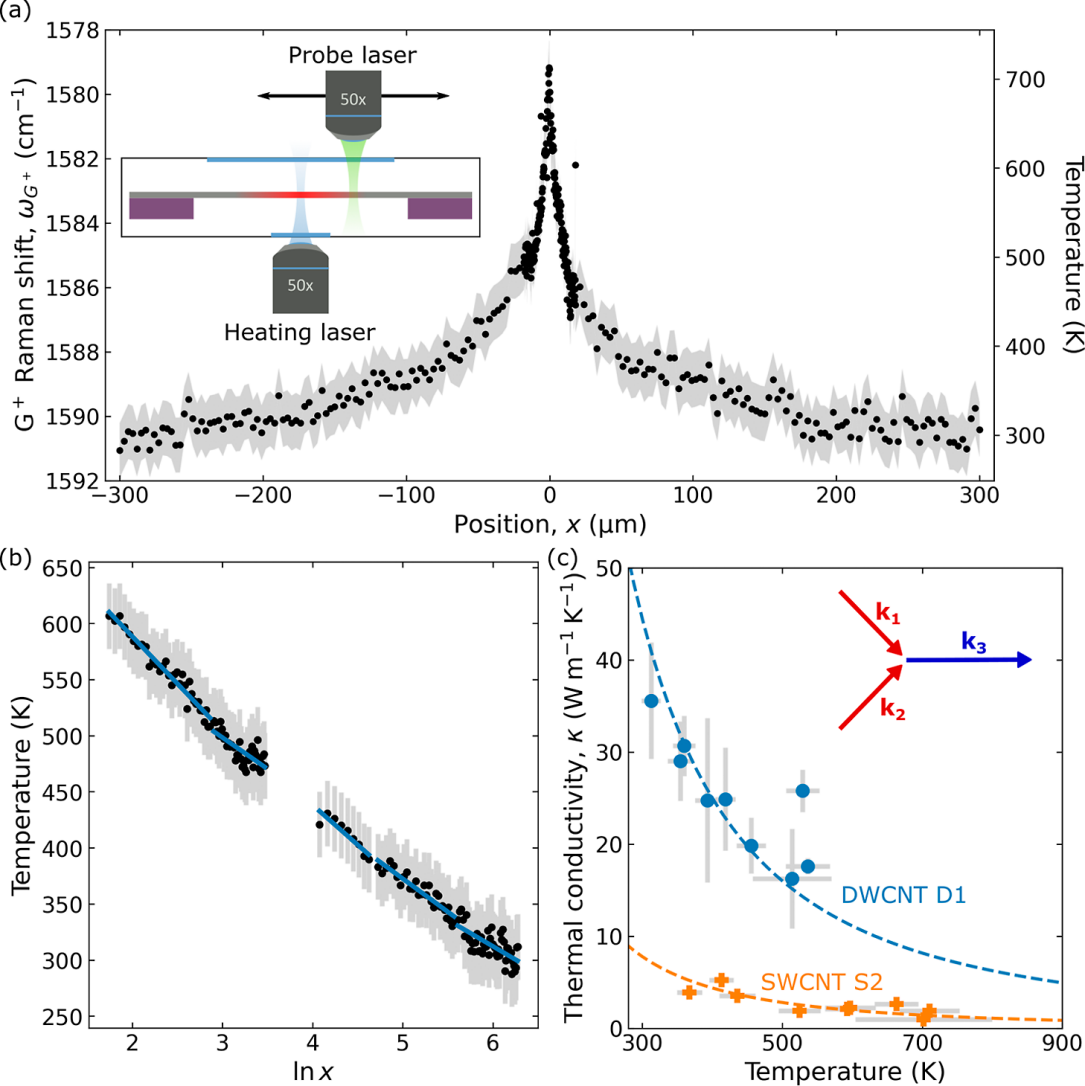
2LRT of CNT films. (a) Spatial dependence of the temperature profile obtained by 2LRT of the DWCNT pellicle (D1). The inset shows the schematic of the experimental setup. We convert the G^+^ mode frequency to temperature using the calibration obtained in Figure 3 (see also the main text). (b) Average temperature from positive and negative *x* positions, shown in a log scale. Here, we obtain the effective thermal conductivity (κ_eff_) by using eq 2. By binning *x* regions, we obtain the temperature-dependent value, κ_eff_(*T*). In (a,b), the gray error bars arise from point-to-point variations in the G^+^ peak position, see Note S1. This corresponds to an uncertainty in temperature of Δ*T* = ± 30 K. (c) κ_eff_(*T*) for the SWCNT S2 (orange) and DWCNT D1 (blue) samples. The dashed lines follow a *T*^–2^ power law, typical for three-phonon (anharmonic) scattering processes. The inset illustrates a three-phonon process involving two acoustic and optical phonons with momenta *k*_1,2,3_.

Using two-laser Raman thermometry (2LRT), we obtained the spatial temperature profile shown in Figure 4. The temperature decays from 700 K close to the heating laser spot to 300 K when the heating spot and the temperature probing spot are separated by a few 100 μm. This spatial profile reveals the steady-state thermal profile in which heat diffuses radially away from the central hot spot. Intuitively, a material with a higher thermal conductivity would have a longer decay length and result in a broader profile.

In the 2LRT model, we extract the spatial dependence of the temperature close to the tails of the decay (|*x*| = 200–300 μm). This describes the thermal transport close to ambient temperature (300 K). Using eq 2, we obtain a thermal conductivity for the DWCNT D1 sample of κ = 44.0 ± 9.9 W m^–1^ K^–1^, which is in good agreement with the values obtained from 1LRT. Here, the clear advantages of the 2LRT technique are that we obtain the thermal conductivity close to room temperature and that we make no assumptions on geometry as we directly visualize the steady-state temperature profile, which directly relates to the thermal conductivity.

**Figure 5:**
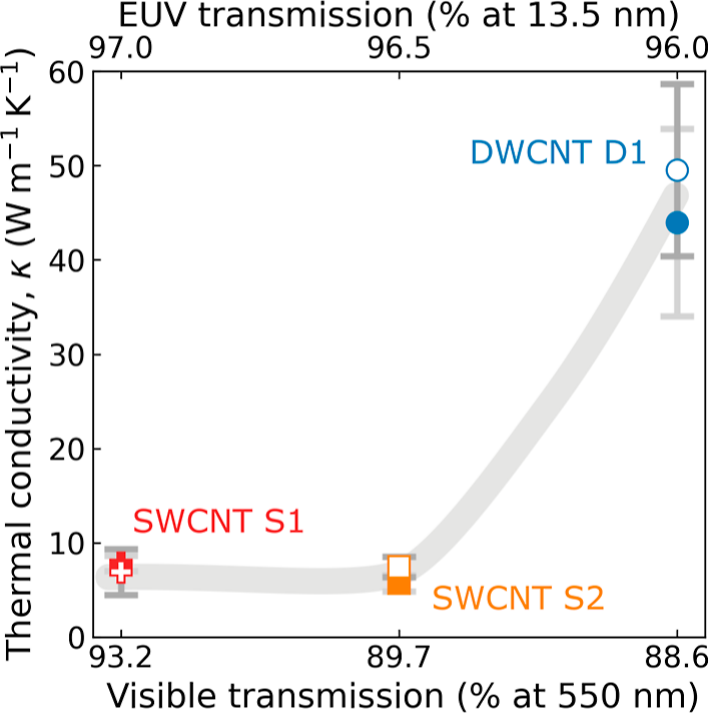
Effective thermal conductivity of free-standing CNT films measured using 1LRT (open symbol) and 2LRT (closed symbol) techniques. The top and bottom *x*-axes show the EUV and visible (λ = 550 nm) transmission of each sample, respectively. The gray line is a guide to the eye. The DWCNT D1 sample has a much higher effective thermal conductivity due to the additional wall that provides additional heat-carrying phonon modes and greater protection against defects.

Having established that both techniques produce similar values of thermal conductivity, we now investigated the different samples. First, we note that the thermal conductivity that we obtain is an *effective* thermal conductivity of the composite CNT films, κ_eff_. Later, we corrected for the porosity and extracted a *skeleton* thermal conductivity of the CNT networks themselves, which we will call κ_CNT_. The effective thermal conductivity (κ_eff_) for the investigated CNT samples, which ranges from 6 to 50 W m^–1^ to K^–1^, is plotted in Figure 5. In Table S1, we report the optical and thermal properties of each sample. The effective thermal conductivities of these CNT networks are 2 orders of magnitude lower than those for isolated CNTs due to the presence of voids and intertube junctions that restrict the thermal transport in the CNT network by introducing boundary resistance.^33,36,38,39^ These junctions also act as filters of phonons with mean free paths greater than the distance between the junctions, greatly reducing the cumulative contribution to κ.^39^

For SWCNT S1, κ_eff_ = 6.9 ± 2.4 W m^–1^ K^–1^ from 1LRT and κ_eff_ = 7.8 ± 0.8 W m^–1^ K^–1^ from 2LRT. These values are comparable to those of SWCNT S2, with κ_eff_ = 7.4 ± 1.1 W m^–1^ K^–1^ from 1LRT and κ_eff_ = 5.7 ± 0.9 W m^–1^ K^–1^ from 2LRT. This is expected as both are SWCNT samples, and the 1LRT and 2LRT fit to the data accounts for differences in thickness and in visible transmission.

Interestingly, the DWCNT D1 film has the largest effective thermal conductivity with κ_eff_ = 49.5 ± 9.1 W m^–1^ K^–1^ from 1LRT and κ_eff_ = 44.0 ± 9.9 W m^–1^ K^–1^ from 2LRT. This is roughly seven times higher than that of SWCNT S1 of similar thickness and EUV transmission, which suggests that the difference in visible transmission alone, which differs only by a few percent (see Note S4), cannot explain the enhanced thermal transport. Indeed, DWCNTs have an additional wall that provides both a parallel conduction channel for thermal transport and robust protection against defects that occur in a single wall. In addition to the intertube junctions, phonon-defect scattering also filters out medium and long mean free path phonons further reducing the effective thermal conductivity.^61^ The lower relative intensity of the D peak in Figure 2 supports the interpretation of a lower defect density in the DWCNT sample.

Having identified the different values of effective thermal conductivity in the samples, we now investigated the temperature dependence of their thermal properties. Typically, the effective thermal conductivity changes with temperature. The reduced phonon population at low temperatures and increased phonon–phonon scattering at high temperatures suppress thermal transport. Here, we evaluate the effective thermal conductivity as a function of temperature above ambient conditions. Rather than only extracting the slope of ∂*T*/∂ ln (*r*) close to 300 K, we bin the data into distinct temperature regimes, Figure 4b. We obtained the thermal conductivity using eq 2 and the gradient of a linear fit to each bin, taking the temperature from the mean values.

Figure 4c shows the temperature-dependent effective thermal conductivity, κ_eff_(*T*), for the DWCNT films D1 and SWCNT S2 films. In both cases, κ_eff_(*T*) is largest close to 300 K before reducing at higher temperatures. The dashed lines show a temperature dependence of the form κ_eff_(*T*) ∝ *T*^–2^, which has previously been reported for individual SWCNTs^23^ and is ascribed to second-order three-phonon scattering processes involving two acoustic phonons and one optical phonon, as shown schematically in the inset. Previous studies reported that the junction thermal conductance is independent of temperature.^39^ This result implies that while the intertube junctions limit the overall thermal resistance, the temperature dependence arises from the remaining conduction channels and therefore reflects the intrinsic properties of the CNTs themselves. Moreover, our observation of a relatively strong temperature dependence and relatively high effective thermal conductivity of the CNT network suggests that defects do not play a significant role. This is consistent with our Raman spectra, which show very small D peaks (see Figure 2).

We will first discuss the effective conductivity of the entire CNT film including voids κ_eff_ and then focus on the skeleton conductivity of the CNTs without the voids κ_CNT_. The effective thermal conductivity of both SWCNT films measured in this work falls into the lower range of literature values for SWCNT films (κ_eff_ ≈ 2–200 W m^–1^ K^–1^),^24,30,62,63^ reflecting the random orientation of individual CNTs within these networks. Indeed, the alignment of SWCNTs by filtration and a high magnetic field increases the thermal conductivity substantially (κ_eff_ = 210 W m^–1^ K^–1^).^24^ We note that in most studies, the CNT films are supported on a substrate. Disentangling the intrinsic and extrinsic factors contributing to thermal transport is not trivial. Therefore, by using free-standing CNT films in this study, we directly probe their intrinsic thermal properties.

Literature reports of the effective thermal conductivity for multiwall carbon nanotube (MWCNT) films typically range from κ = 0.2 W m^–1^ K^–1^, for compressed random networks with a volume fraction of 0.19,^38^ to κ = 50 W m^–1^ K^–1^ measured in the direction parallel to aligned MWCNTs.^25^ For the former, weak bonding between nanotubes impedes thermal transport across nanotube junctions. Interestingly, our results on DWCNT D1 reveal a higher thermal conductivity (κ_eff_ ≈ 50 W m^–1^ K^–1^) than that in typical randomly oriented films of single- or multi-walled CNTs (κ_eff_ ≈ 0.2–2 W m^–1^ K^–1^).^30,31,38,63^ Hence, aligning DWCNTs within the network should offer further improvements in the thermal conductivity.

It is important to note that the thermal conductivities that we have obtained using 1LRT and 2LRT are the effective thermal conductivities of composite films formed by CNTs and voids. In order to obtain the conductivity of the CNT network itself, we follow ref (64) and first quantify the porosity of the films. From image analysis (see Note S2), we obtain porosities ϕ of 0.301 and 0.254 for the DWCNT and SWCNT, respectively. We then use the Maxwell-Garnett effective medium model to obtain the volume correction factor , which is 0.538 and 0.595 for the DWCNT and SWCNT, respectively. Finally, we extract the thermal conductivity of the carbon nanotube network κ_CNT_ using κ_eff_ = < cos θ > κ_CNT_*V*_CNT_, where < cos θ> = 1/3 represents the angular distribution between the CNT axis and direction of heat flow in a randomly oriented film. We determine the CNT skeleton conductivity to be κ_CNT_ = 257 W m^–1^ K^–1^ and κ_CNT_ = 35 W m^–1^ K^–1^ for the DWCNT (D1) and both SWCNT samples (S1/S2), respectively (see Note S2). These values are relevant for comparison with composite materials containing CNTs.^65^

## Conclusions

In conclusion, we experimentally studied thermal transport in free-standing CNT films using Raman thermometry at temperatures between 300 and 700 K. By calibrating the softening of a suitable Raman mode (G^+^) with temperature, we map the thermal field and extract the temperature-dependent thermal conductivity, κ(*T*). At 300 K, κ was measured to be 49.5 W m^–1^ K^–1^ for the DWCNT films and 6.9–7.4 W m^–1^ K^–1^ for the SWCNT films. The significantly higher value for DWCNT films arises from the reduced phonon-defect scattering and additional conductance channel due to the presence of the second wall in the structure of the nanotube. In addition, aligning the CNTs should further increase this value. At higher temperatures, the thermal conductivity decreases from the value at 300 K following the power law: κ(*T*) ∝ *T*^–2^. This dependence is due to second-order three-phonon scattering processes. Interestingly, although the presence of junctions between neighboring nanotubes impedes the overall thermal conductivity, the underlying thermal transport mechanism has the same dependence as that for individual nanotubes. In future work, it will be interesting to study in more detail how the effective thermal conductivity of the CNT network is related to the microscopic structure of the network, for example, considering the number of tube–tube interconnections.

The efficient heat dissipation of free-standing CNT films is attractive for EUV pellicle applications, where a highly EUV-transparent pellicle material must withstand the heat associated with EUV exposures. This study of the thermal properties of both SWCNT and DWCNT free-standing films enhances our understanding of how CNTs can advance various applications. For example, the relatively high effective thermal conductivity of the DWCNT film containing the CNT network and voids (49.5 W m^–1^ K^–1^) and of the CNT network alone (257 W m^–1^ K^–1^) allows for efficient thermal management in flexible devices. The electrical and thermal properties of CNT films are also relevant for applications such as interconnects in integrated circuits, where traditional materials such as copper perform significantly worse at reduced dimensionality.

## Data Availability

All data are available in the main text or Supporting Information and can be obtained upon reasonable request from the corresponding author.

## Supporting Information Available

Distribution of G peak position across the suspended film, effect of porosity on thermal conductivity, thickness of the CNT networks, and optical and thermal properties of CNT networks (PDF)
