# Supplementary material for: Enhanced Thermal Conductivity of Free-Standing Double-Walled Carbon Nanotube Networks
Source: ACS Appl Mater Interfaces. 2023 Oct 27;15(44):51876–84. doi: 10.1021/acsami.3c09210 (PMC10636713; doi:10.1021/acsami.3c09210)
Supplement: Supplementary file 1 — am3c09210_si_001.pdf [file am3c09210_si_001.pdf]

Supporting Information for:

Enhanced thermal conductivity of free-standing double-walled carbon nanotube networks

Jake Dudley Mehew,[†] Marina Y. Timmermans,[‡] David Saleta Reig,[†] Stefanie Sergeant,[‡] Marianna Sledzinska,[†] Emigdio Chávez-Ángel,[†] Emily Gallagher,[‡] Clivia M. Sotomayor Torres,[†,¶] Cedric Huyghebaert,[‡,§] and Klaas-Jan Tielrooij[*,†,∥]

†*Catalan Institute of Nanoscience and Nanotechnology (ICN2), BIST and CSIC, Campus UAB, 08193 Bellaterra (Barcelona), Spain*

‡*imec vzw, Kapeldreef 75, 3001 Leuven, Belgium*

¶*ICREA, Passeig Lluís Companys 23, 08010 Barcelona, Spain*

§*Present address: Black Semiconductor gmbh, 52072 Aachen, Germany*

∥*Department of Applied Physics, TU Eindhoven, Den Dolech 2, Eindhoven, 5612 AZ, The Netherlands.*

E-mail: klaas.tielrooij@icn2.cat

Supplementary Note 1: Distribution of G peak position across suspended film.

The grey bars in Figure 4a represent the estimated error associated from point-to-point variations in G^+ peak position, obtained from 100×100 μm^2 Raman maps in five regions of the suspended DWCNT film, see Supp. Fig. 1. These variations arise from non-thermal effects such as strain and doping. Overall, the sample is highly uniform as evident by the similar mean peak positions (μ) and standard deviations (σ). Using the temperature calibration from Figure 2, we find an uncertainty in temperature of $\Delta T = \pm 30$ K.

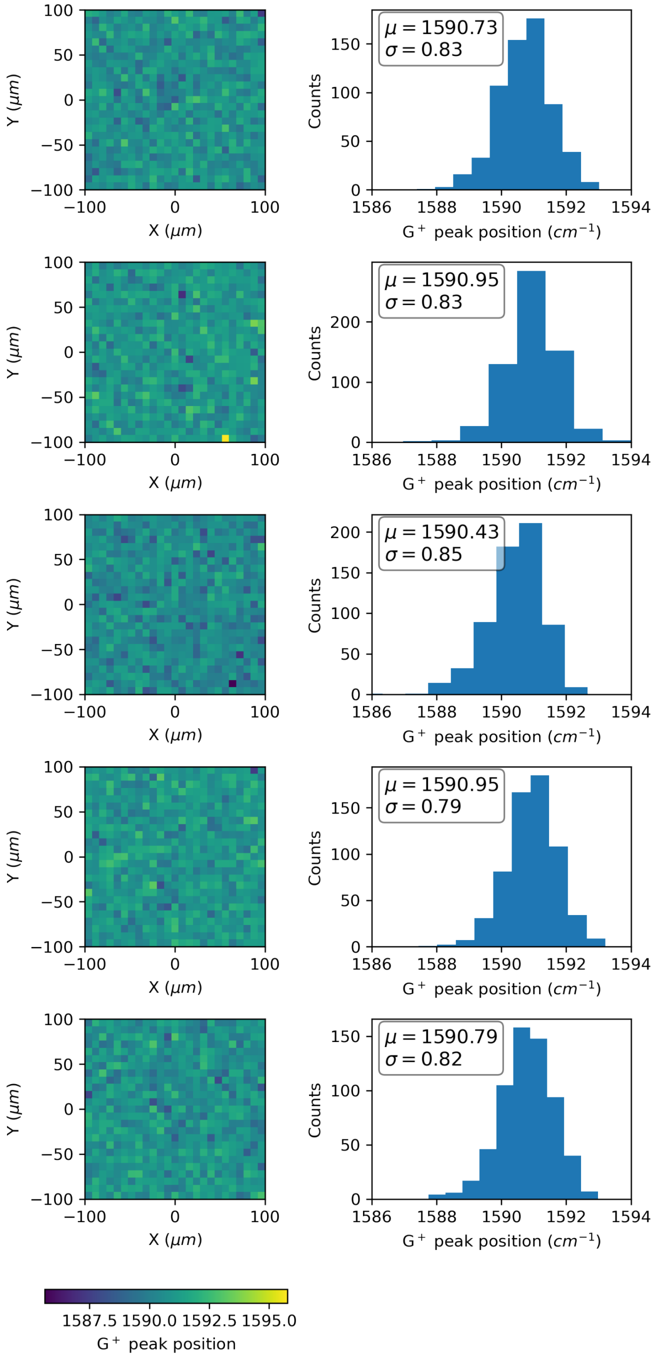

Supplementary Fig. 1: 100 μm by 100 μm spatial maps of G^+ peak position (left) and corresponding histogram (right) for the DWCNT D1 sample. Each map is acquired in a different region of the suspended film. The variation in G^+ peak position is due to non-thermal effects such as strain and doping, which leads to an uncertainty in evaluating local temperature in 2LRT.

Supplementary Note 2: Effect of porosity on thermal conductivity.

The models used to calculate the thermal conductivity from one and two laser Raman thermometry assume a continous medium. However, due to the porosity of the pellicle this results in an underestimation of the thermal conductivity of the CNT network.

In order to correct for this, we estimate the porosity using ImageJ and calculate a volume correction factor using the Maxwell-Garnett effective medium model:[1]

$$V = \frac{1-\phi}{1+\phi}, \tag{1}$$

where the porosity (ϕ) gives the volume correction factor (V). In general, the effective thermal conductivity of the medium (κ_{eff}) is given by:[2,3]

$$\kappa_{\mathrm{eff}} = \left\langle \cos^2\theta \right\rangle \kappa_{\mathrm{CNT}} V_{\mathrm{CNT}}, \tag{2}$$

where κ_{CNT} and V_{CNT} are the thermal conductivity and volume fraction of the CNTs, respectively. $\langle \cos^2\theta \rangle$ represents the distribution of angles between the CNT axis and heat flow direction, which is $\approx 1/3$ for a randomly orientated sample.

Supplementary Figure 2 shows high-resolution SEM images of the DWCNT (a) and SWCNT (b) networks. By applying a threshold to these images (c, d), we identify two distinct regions: those containing CNTs (black) and those without (white). The area fraction of theses two regions gives an estimate of the porosity which is 0.301 and 0.254 for the DWCNT and SWCNT networks respectively. From Equation 1, we obtain volume fractions of 0.538 and 0.595 respectively.

Inputting the volume fractions in Eq. 2, we find that the thermal conductivity of the CNT networks itself (κ_{CNT}) is approximately 5−5.5 times greater than the measured effective value. Therefore, the estimated thermal conductivities for the DWCNT and both SWCNT

networks are ~ 257 W m^{-1} K^{-1} and ~ 35 W m^{-1} K^{-1}.

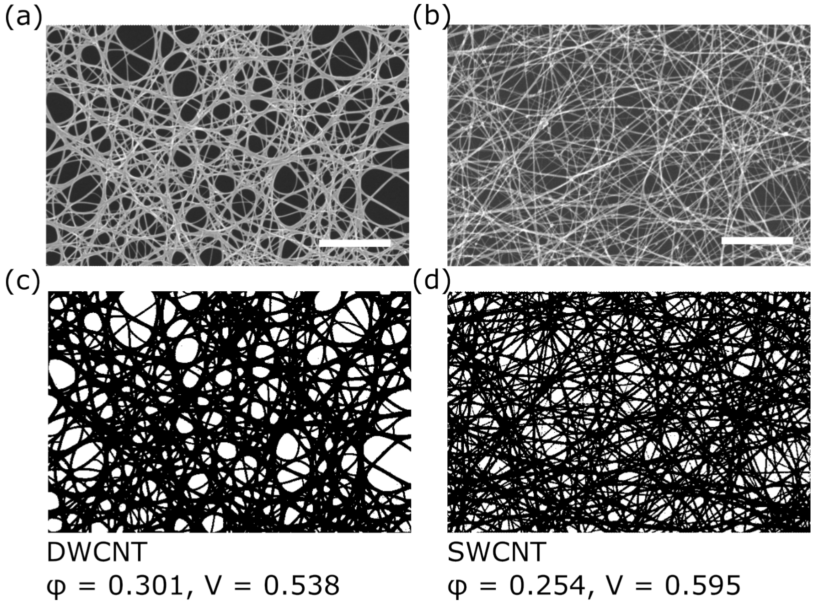

Supplementary Fig. 2: SEM images of the DWCNT (a) and SWCNT (b) networks. (c) and (d) show the corresponding threshold images used to calculate the porosity (ϕ) and volume fraction (V). The scale bar is 500 nm.

Supplementary Note 3: Thickness of the CNT networks.

Supplementary Figure 3 shows the thickness of the CNT networks obtained using atomic force microscopy. We estimate the thickness after scratching a small region of the film that is supported on the silicon frame. By scanning an area of 40×20 μm centred on the scratch (left column), we obtain the height distribution (right column). Here we identify two peaks, one corresponding to the substrate and the other to the CNT network. Finally the thickness is taken from the average of three maps for each sample.

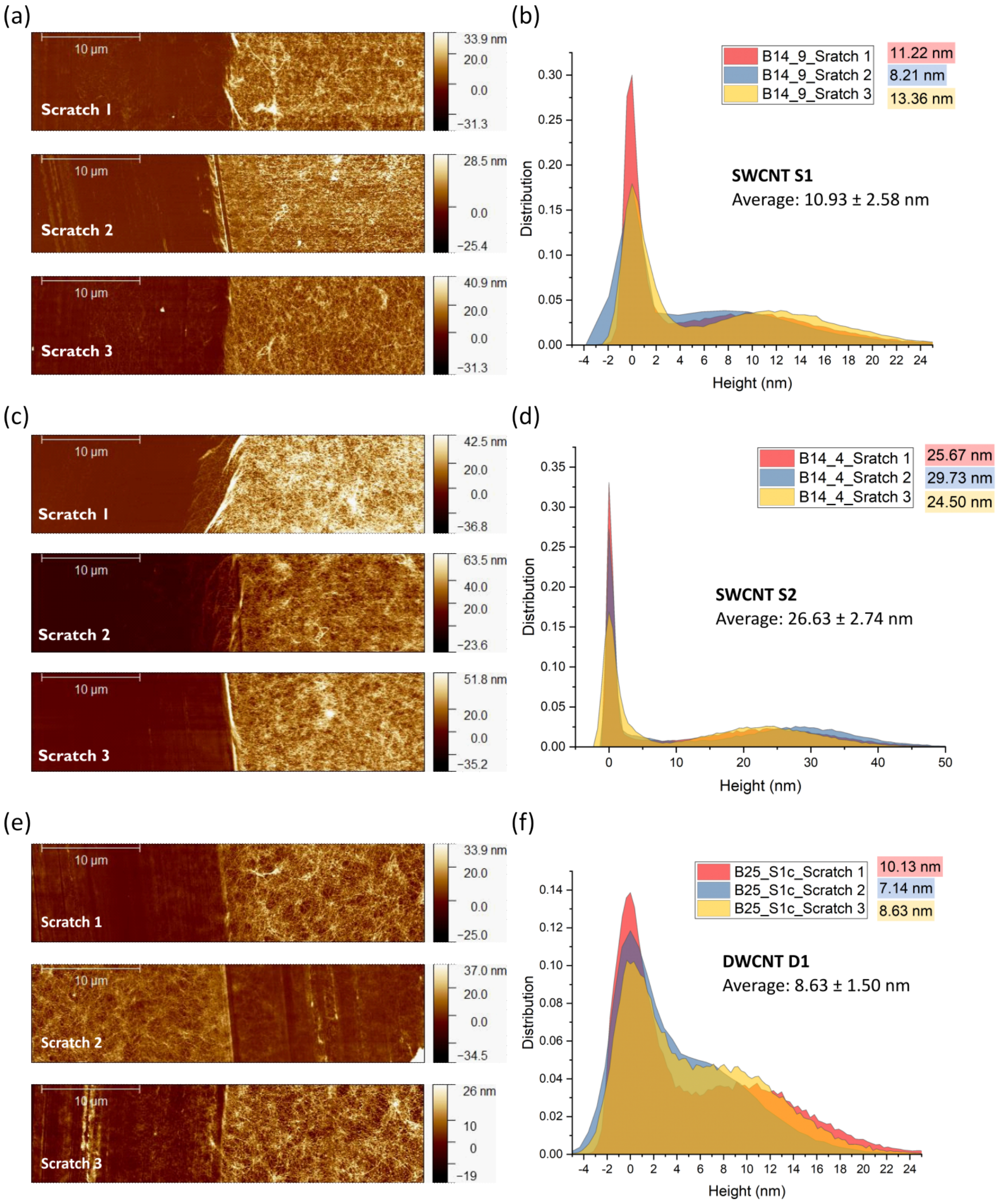

Supplementary Fig. 3: AFM measurements of the thickness of the CNT networks on the silicon support frame. By scratching the film (a,c,e), we obtain the height distribution of the CNT network (b,d,f). The reported thickness is the average of the central point of the three distributions for each sample.

Supplementary Note 4: Optical and thermal properties of CNT networks.

We determined the visible transmission of the CNT films using a Shimadzu UV-Vis spectrophotometer (see Supplementary Figure 4), and obtained the absorption by taking into account an estimated 1% of absorption at 532 nm, based on Ref.[4] We obtained the EUV transmittance at 13.5 nm using a lab-based tool for spectroscopic EUV reflectometry at the PTB metrology institute.[5] The Supplementary Table summarizes the optical and thermal properties of the suspended CNT pellicle films. The thermal conductivity (κ_{eff}) is reported for both one-laser and two-laser Raman Thermometry (1LRT and 2LRT respectively). κ_{CNT} is the porosity-corrected value for the CNT networks. The power and temperature dependence of the G^+ Raman mode shift are χ_P and χ_T respectively.

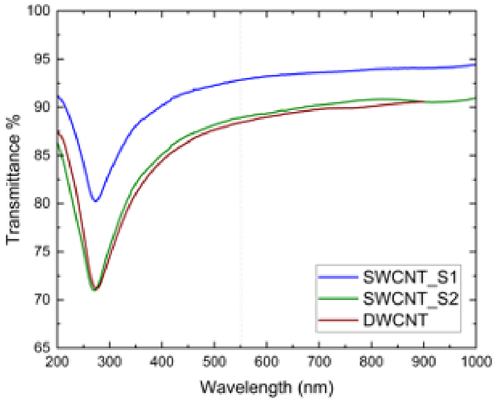

Supplementary Fig. 4: UV-visible transmission measurement of the CNT films.

Supplementary Table S1: Summary of optical and thermal properties of the suspended CNT pellicle films.

	SWCNT S1	SWCNT S2	DWCNT D1
Thickness (nm)	10.93 ± 2.58	26.63 ± 2.74	8.63 ± 1.50
Transmission at 532nm	92%	89%	88%
Absorption at 532 nm	7%	10%	11%
EUV transmission	97%	96.5%	96%
κ_{eff} 1LRT ($\text{Wm}^{-1}\text{K}^{-1}$)	6.89 ± 2.43	7.44 ± 1.08	49.53 ± 9.12
κ_{eff} 2LRT ($\text{Wm}^{-1}\text{K}^{-1}$)	7.83 ± 0.81	5.72 ± 0.86	43.98 ± 9.93
κ_{CNT} ($\text{Wm}^{-1}\text{K}^{-1}$)	35 ± 4	35 ± 4	257 ± 52
χ_P ($\text{cm}^{-1}\mu\text{W}^{-1}$)	-0.1545 ± 0.0054	-0.0812 ± 0.0034	-0.0578 ± 0.0019
χ_T ($\text{cm}^{-1}\text{K}^{-1}$)	-0.0139 ± 0.0036	-0.0192 ± 0.0018	-0.0295 ± 0.0015

References

(1) Lee, J.; Lee, W.; Wehmeyer, G.; Dhuey, S.; Olynick, D. L.; Cabrini, S.; Dames, C.; Urban, J. J.; Yang, P. Investigation of Phonon Coherence and Backscattering Using Silicon Nanomeshes. Nature Communications **2017**, 8, 14054.

(2) Nan, C.-W.; Birringer, R.; Clarke, D. R.; Gleiter, H. Effective Thermal Conductivity of Particulate Composites with Interfacial Thermal Resistance. Journal of Applied Physics **1997**, 81, 6692–6699.

(3) Prasher, R. S.; Hu, X. J.; Chalopin, Y.; Mingo, N.; Lofgreen, K.; Volz, S.; Cleri, F.; Keblinski, P. Turning Carbon Nanotubes from Exceptional Heat Conductors into Insulators. Physical Review Letters **2009**, 102, 105901.

(4) Zhigunov, D. M.; Shilkin, D. A.; Kokareva, N. G.; Bessonov, V. O.; Dyakov, S. A.; Chermoshentsev, D. A.; Mkrtchyan, A. A.; Gladush, Y. G.; Fedyanin, A. A.; Nasibulin, A. G. Single-walled carbon nanotube membranes as non-reflective substrates for nanophotonic applications. Nanotechnology **2021**, 32, 095206.

(5) Bahrenberg, L.; Danylyuk, S.; Loosen, P.; Brose, S.; Stollenwerk, J.; Gallagher, E.; Pollentier, I.; Timmermans, M. Characterization of pellicle membranes by lab-based spectroscopic reflectance and transmittance measurements in the extreme ultraviolet. International Conference on Extreme Ultraviolet Lithography 2017. 2017; p 57.
